# Reverse transcriptase domain sequences from tree peony (*Paeonia suffruticosa*) long terminal repeat retrotransposons: sequence characterization and phylogenetic analysis

**DOI:** 10.1080/13102818.2014.925312

**Published:** 2014-07-24

**Authors:** Da-Long Guo, Xiao-Gai Hou, Tian Jia

**Affiliations:** ^a^ College of Forestry, Henan University of Science and Technology, Luoyang, Henan, P. R. China; ^b^ College of Agriculture, Henan University of Science and Technology, Luoyang, Henan, P. R. China

**Keywords:** tree peony, Ty1-*copia* retrotransposons, sequence heterogeneity, reverse transcriptase

## Abstract

Tree peony is an important horticultural plant worldwide of great ornamental and medicinal value. Long terminal repeat retrotransposons (LTR-retrotransposons) are the major components of most plant genomes and can substantially impact the genome in many ways. It is therefore crucial to understand their sequence characteristics, genetic distribution and transcriptional activity; however, no information about them is available in tree peony. Ty1-*copia*-like reverse transcriptase sequences were amplified from tree peony genomic DNA by polymerase chain reaction (PCR) with degenerate oligonucleotide primers corresponding to highly conserved domains of the Ty1-*copia*-like retrotransposons in this study. PCR fragments of roughly 270 bp were isolated and cloned, and 33 sequences were obtained. According to alignment and phylogenetic analysis, all sequences were divided into six families. The observed difference in the degree of nucleotide sequence similarity is an indication for high level of sequence heterogeneity among these clones. Most of these sequences have a frame shift, a stop codon, or both. Dot-blot analysis revealed distribution of these sequences in all the studied tree peony species. However, different hybridization signals were detected among them, which is in agreement with previous systematics studies. Reverse transcriptase PCR (RT-PCR) indicated that Ty1-*copia* retrotransposons in tree peony were transcriptionally inactive. The results provide basic genetic and evolutionary information of tree peony genome, and will provide valuable information for the further utilization of retrotransposons in tree peony.

## Introduction

Retrotransposons are the most abundant class of mobile genetic elements in plants. They transpose via reverse transcribed RNA intermediates and integrate into new locations within the host genome by means of a `‘copy-and-paste’ mechanism.[1] This mechanism can cause the copy number of the retrotransposons to increase and create stable insertion mutations in the host genome.[2] Retrotransposons are divided into two principal groups: long terminal repeat (LTR) retrotransposons and non-LTR retrotransposons based on the presence or absence of an LTR sequence.[3] The LTR retrotransposons can be further sub-divided into two groups, the Ty1-*Copia* group and the Ty3-*Gypsy* group, on the basis of their degree of sequence similarity as well as the order of their genes.[3]

Retrotransposons can not only greatly increase the plant genome size because of the replicative mode of transposition, but can also generate mutations by inserting new copies within or near genes.[4] However, because of the presence of stop codons, frame shifts, and deletions, most retrotransposons appear to be non-functional.[3] Therefore, there is the possibility to study the transposition history of the retrotransposons for understanding their evolution and mutations in the host genome. Ty1-*copia* retrotransposons are present throughout the plant kingdom, ranging from algae to bryophytes, gymnosperms and angiosperms [5] and are usually present in plant genomes in a high copy number and have high degrees of heterogeneity and insertional polymorphism. These characteristics, shared by many LTR retrotransposons, make the excellent bases for marker systems.[6,7,8] To date, Ty1-*copia* retrotransposons have been utilized as molecular markers for genetic research in diverse plants due to their high copy numbers and heterogeneity in plant genomes.[8]

Tree peony (*Paeonia suffruticosa* Andrews.) belongs to the *Moutan* subfamily of the genus *Paeonia*, Paeoniaceae. Tree peonies are known as ‘the king of flowers’ due to their rich plenty of horticultural varieties, great ornamental and medicinal value in China. It has been grown for approximately 1400 years in China.[9] Approximately 2000 cultivars of tree peonies are grown throughout the world, and more than 1000 cultivars are found in China.[10]

However, with the rapid increase in the numbers of species or infra-specific taxa in *Paeonia* sect. *Moutan*, new technology or new information is needed for tree peony taxonomic assessment.[11] It is proved that retrotransposons have contributed to increasing the genome size in the plant kingdom, and play an important role in the evolution of the genome.[3,12] At the same time, tree peonies have abundant genetic diversity; for example, there are many flower colours in tree peony cultivars (white, red, purple, yellow, secondary and compound colours) and diverse flower shapes (single, lotus, chrysanthemum, rose, crown, globular and others).[13] There are also four cultivar groups based on geographic locations in China: Zhongyuan, Xibei, Xinan and Jiangnan.[14] However, it is unclear how has this genetic diversity formed. Researches in other plants have shown that retrotransposons have played a major role in the formation of colour or other traits.[4,15] That is why, based on the characteristics and function of plant retrotransposons and considering the issues mentioned above in tree peony, the study of retrotransposons may be helpful for the elucidation of tree peony species and cultivars evolution, the formation mechanism of genetic diversity and important agronomic traits.

It has been found that a pair of degenerate oligonucleotide primers based on two highly conserved domains of reverse transcriptase (RT) can successfully be used for polymerase chain reaction (PCR) amplification of *copia*-like sequence.[1,5,6] To date, Ty1-*copia* retrotransposons have been studied and characterized widely in plants such as barley, tobacco, tomato, potato, rice, maize, strawberry, mungbean, stramonium, maguey, jute, etc.[8,12,16,17] Despite the large number of retrotransposons that have been isolated, there have been no reports of LTR retrotransposons and their phylogenetic classification in tree peony (*P. suffruticosa* Andrews.). Until recently, the scarcity of LTR retrotransposon sequences limited the use of retrotransposon-based molecular marker systems in this species.

In order to characterize the heterogeneous population of the *copia* group of retrotransposons in tree peony, we isolated genomic RT sequences by PCR using primers designed from the conserved domains of the RT region of Ty1-*copia* retrotransposons. The aim of this investigation was to test for the presence of Ty1-*copia*-like sequences in tree peony and related species using a PCR-based approach and investigate their sequence heterogeneity, phylogenetic relationships, genetic distribution and transcriptional activity.

## Materials and methods

### Plant materials and isolation of nucleic acids

The tree peony (*P. suffruticosa*) cultivar ‘Luoyanghong’, and two related species, *P. qiui* Y. L. Pei et D. Y. Hong and *P. ostii* T. Hong et J. X. Zhang., were used for RT sequence isolation. A total of 20 varieties were used for the following dot-blot analysis: five varieties of *P. rockii* from different regions, two varieties of *P. delavayi*, another seven related species of tree peony and six varieties from different cultivar groups of *P. suffruticosa*. Table 1 shows the information about the materials. Genomic DNA was isolated from young leaves by a modified hexadecyltrimethylammonium bromide (CTAB) method as described by Guo et al.[18] Total RNA was isolated as performed by Gai et al.[19]

**Table 1.** List of varieties used in this study.

Code	Variety names	Species	Locality or cultivar group
1	*Paeonia lutea* Delavay ex Franch.	*Paeonia lutea* Delavay ex Franch.	Zhongdian, Yunan
2	*Paeonia delavayi* Franch.	*Paeonia delavayi* Franch.	Lanzhou, Gansu
3	*Paeonia rockii* (S. G. Haw & Lauener) T. Hong & J. J. Li ex D. Y. Hong	*Paeonia rockii* (S.G.Haw & Lauener) T. Hong & J. J. Li ex D.Y.Hong	Shenlongjia, Hubei
4	*P. qiui* Y. L. Pei et D. Y. Hong	*P. qiui* Y. L. Pei et D.Y. Hong	Baokang, Shaanxi
5	*Paeonia jishanensis* T. Hong et W. Z. Zhao	*Paeonia jishanensis* T. Hong et W. Z. Zhao	Lanzhou, Gansu
6	*Paeonia delavayi* var. lutea (Franch.) Finet. Et Gagnep.	*Paeonia delavayi* var. lutea (Franch.) Finet. et Gagnep.	Lanzhou, Gansu
7	*Paeonia rockii* (S.G.Haw & Lauener) T. Hong & J. J. Li ex D. Y. Hong	*Paeonia rockii* (S.G.Haw & Lauener) T. Hong & J. J. Li ex D. Y. Hong	Lintao, Gansu
8	*Paeonia rockii* (S.G.Haw & Lauener) T. Hong & J. J. Li ex D. Y. Hong	*Paeonia rockii* (S. G. Haw & Lauener) T. Hong & J. J. Li ex D. Y. Hong	Zhouqu, Gansu
9	*Paeonia rockii* (S.G.Haw & Lauener) T. Hong & J. J. Li ex D. Y. Hong	*Paeonia rockii* (S.G.Haw & Lauener) T. Hong & J. J. Li ex D. Y. Hong	Baokang, Shaanxi
10	*P. ludlowii* (Stern et Taylor) D. Y. Hong	*P. ludlowii* (Stern et Taylor) D. Y. Hong	Lanzhou, Gansu
11	*Paeonia delavayi* Franch.	*Paeonia delavayi* Franch.	Luoyang, Henan
12	*Paeonia rockii* (S.G.Haw & Lauener) T. Hong & J. J. Li ex D. Y. Hong	*Paeonia rockii* (S.G.Haw & Lauener) T. Hong & J. J. Li ex D. Y. Hong	Wenxiang, Gansu
13	*P. decomposita* Hand.-Mazz.	*P. decomposita* Hand.-Mazz.	Lanzhou, Gansu
14	*P. ostii* T. Hong et J. X. Zhang	*P. ostii* T. Hong et J. X. Zhang	Lanzhou, Gansu
15	Luoyanghong	*P. suffruticosa* Andrews.	Zhongyuan
16	Hongxiuqiu	*P. suffruticosa* Andrews.	Xibei
17	Baiyu	*P. suffruticosa* Andrews.	Jiangnan
18	Lantianyu	*P. suffruticosa* Andrews.	Xibei
19	Yaohuang	*P. suffruticosa* Andrews.	Xibei
20	Shouanhong	*P. suffruticosa* Andrews.	Zhongyuan

### PCR amplification and cloning

The internal domain of the RT gene of Ty1-*copia* retrotransposons was amplified by PCR using the flanking primers corresponding to the peptide sequences TAFLHG (5′ ACNGCNTTYYTNCAY GG 3′) and YVDDML (5′-ARCATRTCRTCNACRTA-3) following the methods of Kumar et al.[1] PCR products from the RT region of tree peony were cloned into the PMD-18T vector, using the original TA Cloning Kit (TaKaRa, Dalian, China). The ligation products were transformed into DH5α competent cells and positive clones were sequenced by Sun-Biotech Co. (Beijing, China).

### Sequence analysis

The nature of cloned sequences was confirmed by performing similarity searches with known retrotransposon sequences from other plants in the National Center for Biotechnological Information (NCBI) database using BLASTN, BLASTX and TBLASTX algorithms. The deduced amino acid sequences of tree peony RT sequences were also compared with the RT sequences of other plants for phylogenetic analysis. Genomic DNA sequences were deposited in the GenBank database and the details of these sequences are given in Table 2. Multiple DNA sequence alignments were carried out using Lasergene 8.0. The Bootstrap neighbour-joining (NJ) tree with the tests for 10,000 replications was generated using MEGA 5.0.[20]

**Table 2.** Accessions of RT sequences isolated from tree peony deposited in GenBank.

Varieties name	Abbreviation used	Accession number
Luoyanghong	PS1	JN203072.1
Luoyanghong	PS2	JN203073.1
Luoyanghong	PS3	JN203074.1
Luoyanghong	PS4	JN203075.1
Luoyanghong	PS6	JN203077.1
Luoyanghong	PS7	JN203078.1
Luoyanghong	PS8	JN203079.1
Luoyanghong	PS9	JN203080.1
Luoyanghong	PS10	JN203081.1
Luoyanghong	PS11	JN203082.1
Luoyanghong	PS12	JN203083.1
Luoyanghong	PS13	JN203084.1
Luoyanghong	PS14	JN203085.1
Luoyanghong	PS15	JN203086.1
Luoyanghong	PS16	JN203087.1
Luoyanghong	PS17	JN203088.1
Luoyanghong	PS19	JN203090.1
Luoyanghong	PS20	JN203091.1
Luoyanghong	PS21	JN203092.1
*Paeonia qiui*	PQ10	JX549046
*Paeonia qiui*	PQ11	JX549047
*Paeonia qiui*	PQ12	JX549048
*Paeonia qiui*	PQ21	JX549049
*Paeonia qiui*	PQ22	JX549050
*Paeonia qiui*	PQ23	JX549051
*Paeonia qiui*	PQ24	JX549052
*Paeonia qiui*	PQ25	JX549053
*Paeonia qiui*	PQ26	JX549054
*Paeonia qiui*	PQ27	JX549055
*Paeonia qiui*	PQ28	JX549056
*Paeonia qiui*	PQ29	JX549057
*Paeonia ostii*	PO302	JX549058
*Paeonia ostii*	PO303	JX549059
*Orobanche ramosa*	OR	DQ376441
*Prunus mume*	PM	DQ494250.1
*Fragaria x ananassa*	FA	GU197836.1
*Epimedium coactum*	EC	GQ852889.1
*Lycium ruthenicum*	LR	GU573476.1
*Eleocharis quinqueflora*	EQ	ADF45720.1

### Dot-blot analysis

Genomic DNA and the heterogeneous PCR products of the RT domains of Ty1-*copia* were used for dot-blot analysis. The 270 bp PCR product was labelled using digoxigenin–deoxyuridine triphosphate (DIG–dUTP) by random primed labelling according to the manufacturer's instructions for the DIG DNA Labeling and Detection Kit (Roche, Germany). Hybridization was carried out at 65 °C for 18 h, with washing conditions as recommended by the manufacturer.

### Reverse transcriptase PCR (RT-PCR)

First-strand cDNA was synthesized from total RNA using RT XL (AMV) (TaKaRa, Daliang, China). PCR was carried out using the same degenerate primers and cycling conditions as above, except that 1.0 μL of first-strand cDNA was used as the template. Meanwhile, genomic DNA (gDNA) was used as a positive control.

## Results and discussion

### Isolation of Tyl-*copia* RT sequences from tree peony genome

**Figure 1.**
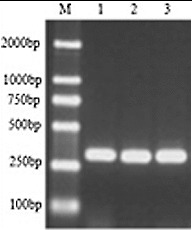
PCR amplification of RT domains of Ty1-*copia* retrotransposons from tree peony genomic DNA, using degenerate primers. Arrow indicates the expected ∼270 bp band. Lane M contains 100 bp DNA ladder. M: DL 2000 Marker, 1: *P. suffruticosa* cv. Luoyanghong, 2: *P. qiui,* 3: *P. ostii*.

Different degenerate primers corresponding to the conserved RT domains were identified by Flavell et al.,[6] Hirochika and Hirochika,[7] Kumar et al. [1] and Voytas et al. [5] in plants. The highly conserved amino acid sequences of enzyme domains have proven to be a useful basis for the design of PCR primers for the purpose of transposon surveys in a number of plant species.[12,21,22] These primers were also used in the preliminary study. However, only the degenerate oligonucleotide primers corresponding to the TAFLHG and YVDDML regions designed by Kumar et al. [1] were successfully used to amplify the conserved RT domains of the tree peony. An amplicon of ∼270 bp was obtained from the tree peony genome of Luoyanghong, *P. qiui* and *P. ostii*, using PCR (Figure 1). This is in agreement with the results of others [17,23] and indicates that Ty1-*copia* retrotransposons were distributed in all the studied tree peony genomes and have a similar length of RT sequences.

### Sequence characterization of tree peony Ty1-*copia* RT clones

Nineteen clones containing 270 bp PCR products from Luoyanghong, twelve clones from *P. qiui* and two clones from *P. ostii*, were randomly selected for sequence analysis. The results showed that 33 clones with RT sequences of Ty1-*copia* group retrotransposons were identified. Their DNA sequences were deposited in the NCBI nucleotide sequence database (Table 2). Homology-based searching (BLASTx and BLASTn) revealed that these putative RT clones have nucleotide sequence similarities to the RT domains of other known plant Ty1-*copia* group retrotransposons (data not shown). Six additional RT sequences previously obtained from other plants (Table 2) which were highly similar to the tree peony putative RT sequences were added for further analysis.

**Figure 2.**
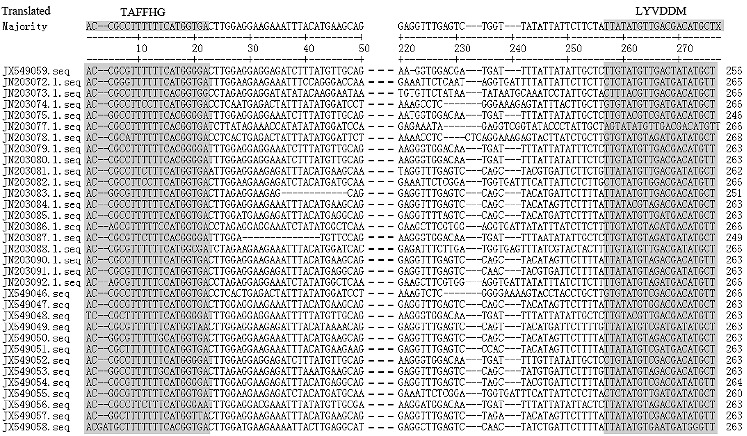
Sequence alignment of the deduced amino acid sequences corresponding to the reverse transcriptase domain (RT) of the Ty1-*copia* group retrotransposons in tree peony. Gaps and stop codons are indicated as (--) and (.), respectively. Numerals on the right are the number of amino acid residues in the sequences. The three shaded boxes indicate the conserved residue of the sequences. The details of RT sequences are given in Table 2.

The putative Tyl-*copia* RT sequences were translated into their amino acids and analysed for the presence of stop codons and frame shifts in their coding regions. Translation of these PCR amplified sequences implied that among the 33 Ty1-*copia* RT sequences, 19 (approximately 58%) sequences contained in-frame stop codon(s) which inferred that these sequences do not have potential function of a RT fragment. The stop-codon-containing sequences are PS1, PS2, PS4, PS6, PS8, PS9, PS10, PS12, PS14, PS16, PS17, PQ10, PQ12, PQ22, PQ25, PQ26, PQ28, PO302 and PO303, in which one to four stop codons were present within coding regions of the retrotransposons (Figure 2). The remaining 14 amplified sequences (approximately 42%) were considered to be partial Ty1-*copia* RT sequences of tree peony with potentially functional RT domain, as they lack any in-frame stop codon. Several of the RT sequences had frame shifts: the 13th amino acid in PS1, the 14th amino acid in PS2, the 18th amino acid in PS4, the 12th amino acid in PS12, the 8th amino acid in PS16, the 14th amino acid in PQ21, the 17th amino acid in PQ26, the 13th amino acid in PO303, the 1st amino acid in PO302, and so on. The age of an element is directly proportional to the number of termination codons incorporated in it since the termination codons are not accumulated in an active element due to its continuous retrotransposition.[24] For example, a study of Ty1-*copia* retrotransposons in strawberry has identified that 5 of 19 RT gene fragments are characterized with stop codons and/or frameshifts,[25] whereas in persimmon 51% of the Ty1-*copia* RT sequences have been shown to have stop codons and/or frameshifts.[26] In this study, more than 58% of Ty1-*copia* RT sequences showed stop codons and/or frameshifts when translated. The percentage is higher than that in other plants, which suggests that there have possibly been frequent activations of tree peony Ty1-*copia* retrotransposons in the history of this species and maybe this could be one of the reasons that have led to the abundant genetic diversity existing in tree peony.

The alignment and the in silico analysis of the deduced amino acid sequences of all 33 sequences revealed the presence of blocks of residues of highly conserved regions among them (Figure 2). They contained characteristic amino acid motifs for the RT gene (5′-TAFF(L)HG; central region, YGLKQ and 3′YVDDM). Most of these sequences showed strong homology to the RT conserved domains of the Ty1-*copia-*like retrotransposons of various plant species.[1] Although the amino acid motifs of few sequences of Ty1-*copia* retrotransposons of tree peony did not show any similarity (Figure 2), the homology-based searches (BLASTn and BLASTx) confirmed these as sequences of Ty1-*copia*-like retrotransposons. These results suggest that the above-mentioned clones represent a portion of the RT domain of the Ty1-*copia* group retrotransposons in tree peony.

Li [14] summarized the previous researches and presented the viewpoint that *P. jishanensis*, *P. rockii*, *P. qiui* and *P. ostii* are the wild ancestor of *P. suffruticosa* based on the morphology and cultivation history, which was further supported by Zhang et al.[11] Hou et al. [27] reported that *P. ostii* had the closest relationships with the Zhongyuan cultivar group of *P. suffruticosa* by eight amplified-fragment-length-polymorphism (AFLP) primer analysis. However, the alignments in our study showed that the sequence similarity between Luoyanghong and *P. qiui* was higher than the one between Luoyanghong and *P. ostii*. The *P. ostii* sequences are more divergent than that of Luoyanghong (*P. suffruticosa*). In fact, eight sequences were obtained from *P. ostii*, but only two were identified as putative Tyl-*copia* RT sequences after Blast search and alignments. This reflects the extreme sequence heterogeneity existing in *P. ostii*. It indicated that the genetic relationship between *P. suffruticosa* and *P. qiui* is closer than that between *P. suffruticosa* and *P. ostii*. This disagreement with Hou et al. [27] may be due to the limited primers used in their study.

### Phylogenetic analysis of tree peony Ty1-copia RT sequences

Due to high variability in the nucleotide sequences, multiple sequence alignments were performed on amino acid sequences, using Lasergene. Within plants, sequence analyses of the RT genes revealed extremely high heterogeneity even in the same species.[28] High level of heterogeneity was found among the 33 peptide sequences, all showing between 10.7% (PQ29 and PO302) and 96.6% (PS13 and PQ23) identity to one another.

**Figure 3.**
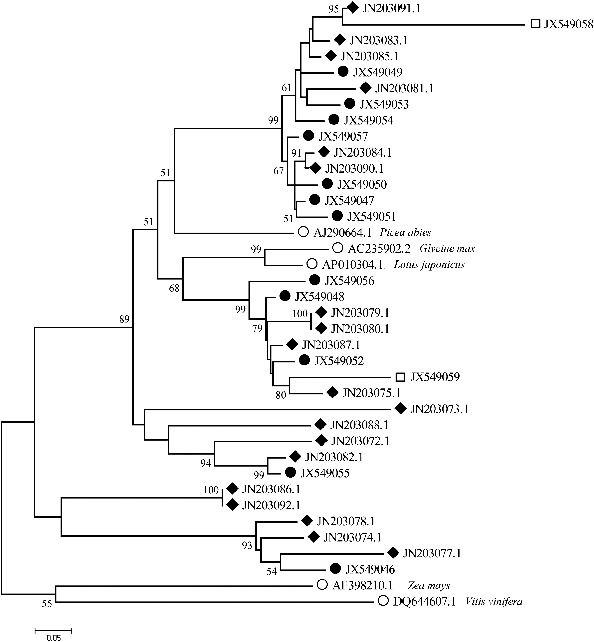
Phylogenetic analysis of the deduced amino acid sequences representing fragments of the RT domain of Ty1*-copia* retrotransposons in tree peony and other plants, using the neighbour-joining method. The numbers in the branches represent bootstrap support for 10000 replicates. The details of the sequence names are given in Table 2.

For comparative purposes, we included in the phylogenetic analysis Ty1-*copia* group RT sequences from other species deposited in the GenBank database (Figure 3). The phylogenetic relationship among the RT sequences of Tyl-*copia* group retrotransposons was represented as a NJ-tree based on P-distance and supported with 10,000 replicates of bootstrapping (Figure 3). The phylogenetic analysis showed that the sequences could be grouped into six distinct families (F1–F6) and that most Tyl-*copia* RT sequences in tree peony were closely related to the representative elements of other plant species. The first family in Figure 3 included five sequences all from Luoyanghong and seemed to be associated with GQ852889.1 isolated from *Epimedium coactum*, DQ376441 from *Orobanche ramosa*, GU573476.1 from *Lycium ruthenicum*, as well as GU197836.1 from *Fragaria x ananassa*. The second family comprised of three sequences from Luoyanghong and three ones from *P. qiui* and did not include any from other plant sequences. The third family was found to be largest, consisting of five sequences from Luoyanghong and six sequences from *P. qiui*. ADF45720.1 from *Eleocharis quinqueflora* was associated with family 3 and its members were very similar to each other, indicating that they were recently duplicated. Family 4 containing one single sequence (PO302) was separated as an independent group because of its long distance to the remaining sequences. Family 5 is the most complicated; it included two sequences from Luoyanghong, two sequences from *P. qiui* and one from *P. ostii* and appeared to be related to DQ494250.1 from *Prunus mume* ABF57057. The RT sequences showed the high diversity of retrotransposons within tree peony. The sequences isolated from different species of tree peony are mixed together and could not be distinguished from each other, indicating that most probably a vertical transmission had happened during the evolution of these retrotransposons in tree peony. Furthermore, when the identified families span species boundaries, it could be suggested that they existed early in plant evolution prior to modern plant species divergence.[29] The divergent families probably represent parallel evolution of groups of sequences from a common ancestor and the result of the phylogenetic analysis showed that the RT sequences from tree peony had high homology with those from other species, such as *Lycium ruthenicum* and *Prunus mume* (Figure 3), which supports a horizontal mechanism of transmission.

The phylogenetic analysis revealed that tree peony Ty1-*copia* group retrotransposon families 4, 5 and 6 possess long branches. On the other hand, the remaining families (1–3) are characterized with short branches. Branch lengths are known to be proportional to sequence divergence and may be considered a result of faster sequence evolution brought about at least partly by the error-prone nature of RT.[29,30] The sequence of PO302 from *P. ostii* and F4 in Figure 3 comprises a unique sequence in a phylogenetic context, with the longest branch, which indicated that *P. ostii* are more primitive than *P. suffruticosa* and *P. qiui*. Clones of Luoyanghong form a large clade composed of closely related sequences, however, sequences of *P. qiui*, especially of *P. ostii* are more variable. These data, combined with other comprehensive analyses,[14] suggest that retroelements could have played a role in differential genome evolution, similar to that in other plant groups.[21]

### Dot-blot analysis

To determine the copy numbers of Ty1-*copia* elements in a plant genome, Southern blot analysis is usually carried out.[21,26,31] At first, we also attempted to conduct Southern blot and designed some primers to obtain probes based on the consensus sequences from alignments. However, the results were not satisfactory, since the signal was too weak. That is why, dot blotting was employed to investigate the existence of Ty1-*copia* elements in the tree peony genome. Dot blotting has been widely used in the analysis of retrotansposon sequences to determine the copy numbers.[23,25,32,33] The heterogenous populations of 270 bp Ty1-*copia* whole PCR products were used as probes for dot-blot hybridization in this study to acquire the relative numbers of *Copia* retrotransposons. However, due to the lack of information about the length of the tree peony genome at present, we could not obtain the specific copy number of *Copia* elements in tree peony.[23,25,32]

**Figure 4.**
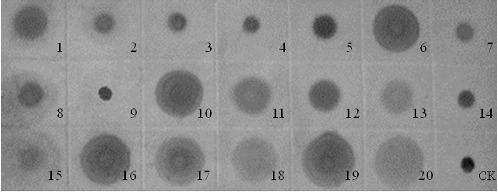
Dot-blot analysis of tree peony genomic DNA. The PCR product of RT sequences was used as a probe. The numbers represented in the materials are given in Table 1.

Dot-blot analysis in this study showed the existence of dispersed copies of the Ty1-*copia* element in tree peony genome because the hybridization signal was detected in all the materials (Figure 4). The detection of multiple bands under both high- and low-stringency hybridization conditions indicates that a large number of sequences homologous to RT are integrated throughout the tree peony genome. However, the intensity of the hybridization signal in different varieties was different (Figure 4), which suggests a presence of a different number of Ty1-*copia* elements in the corresponding genomes. For example, the signals in *P. qiui*, *P. ostii*, *P. rockii* and *P. decomposita* were inferior to those of other species, which indicated lower numbers in them. This result is in accordance with the evolution analysis of Li.[14] However, even in the same species of *P. rockii*, different signals were detected in the varieties collected from various localities. Indeed, it was confirmed that different genetic diversity existed in different populations of *P. rockii*.[9,10] It may be due to the different selection pressures. Another interesting result is that medium signals were detected in *P. ludlowii* and *P. lutea*. Both of them are yellow tree peonies. This indicated their special status in the species of tree peony. Li [14] and Zhang et al. [11] all stated that *P. ludlowii* and *P. lutea* were not involved in the evolution of *P. suffruticosa*. The hybridization intensities of varieties of *P. suffruticosa*, whether it is from Zhongyuan, Xibei, Xinan or Jiangnan, were all stronger than those of *P. qiui*, *P. ostii*, *P. rockii* and *P. decomposita*. This suggested that the copy numbers in *P. suffruticosa* are more than those in *P. qiui*, *P. ostii*, *P. rockii* and *P. decomposita*, which, combined with the results of Li [14] and Zhang et al.,[11] indicates that the retrotransposons of *P. suffruticosa* may have been multiplied in comparison to that of *P. qiui*, *P. ostii*, *P. rockii* or/and *P. decomposita*. This result may possibly indicate that this element is an ancient component of the tree peony genome, introduced before the divergence of the species and conserved during evolution. Of course, more proofs are needed to determine the genetic relationships among the species of tree peony.

### Transcriptional analysis

Plant retrotransposons are usually transcriptionally inactive during the developmental stages to lessen the detrimental effect on the host.[3] The feature of retrotransposons transcriptional activity in tree peony was studied by an RT-PCR approach using degenerate oligonucleotide primers. Total RNA was extracted from leaves of *in vitro* grown tree peony plants, and it was reverse transcribed to obtain the corresponding cDNA. However, no product corresponding to the expected size of Ty1-*copia* retrotransposons was amplified from cDNAs, while the expected product was amplified from the control (gDNA). The results indicated that Ty1-*copia* retrotransposons were transcriptionally inactive in tree peony.

These results could be considered as a first step towards understanding the performance of Ty1-*copia* retrotransposons in tree peony and more work is needed in future in order to expand our knowledge about the activity of Ty1-*copia* retrotransposons and to utilize them in genetic analysis of tree peony. The isolation of full-length Ty1-*copia* retrotransposons and the development of molecular markers based on the retrotransposons of tree peony are ongoing. 
